# Supplementary material for: Theoretical study of ArcB and its dimerization, interaction with anaerobic metabolites, and activation of ArcA
Source: PeerJ. 2023 Oct 13;11:e16309. doi: 10.7717/peerj.16309 (PMC10578306; doi:10.7717/peerj.16309)
Supplement: Supplemental Information 1 [file peerj-11-16309-s001.pdf]

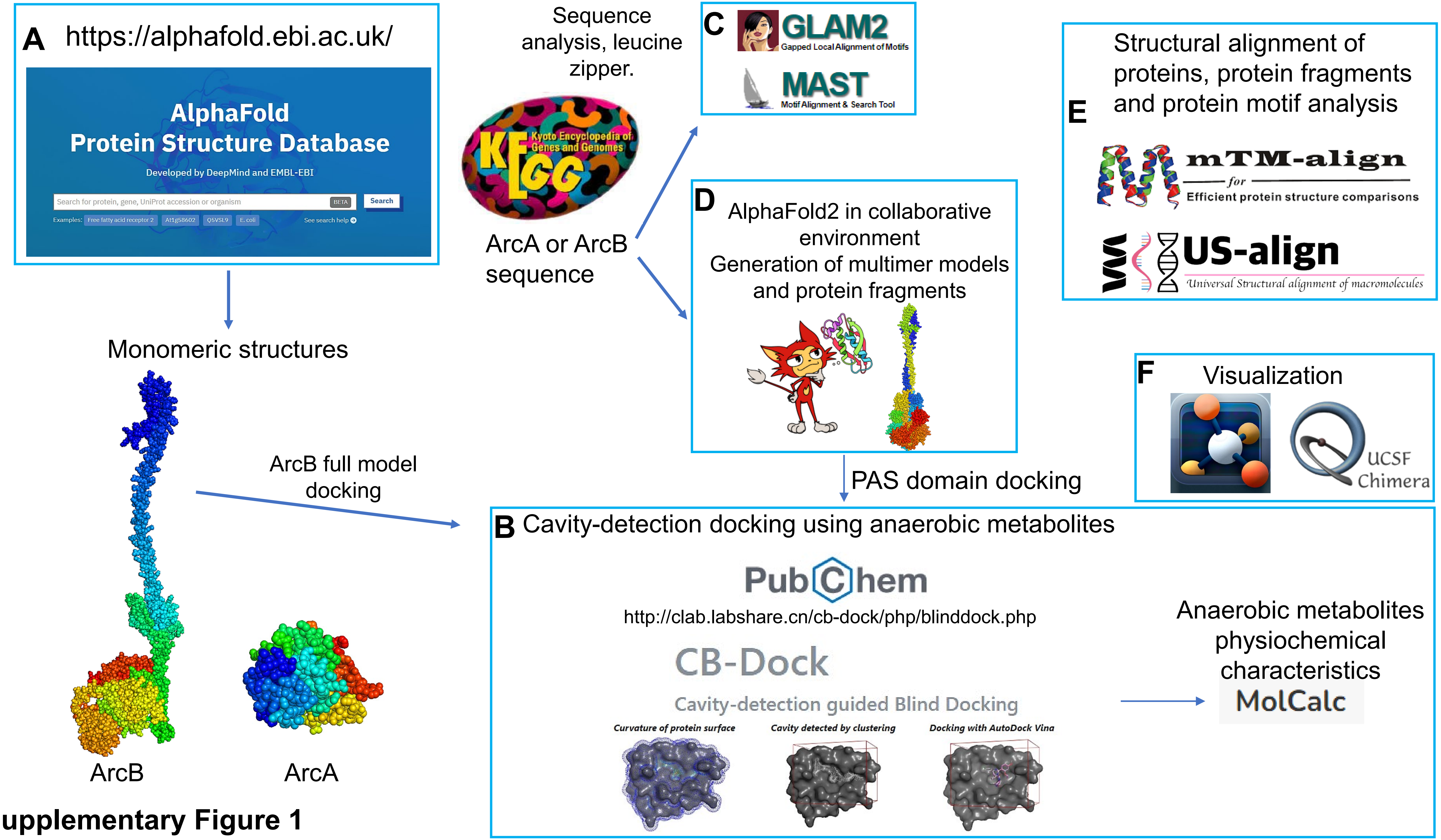

Supplementary Figure 1

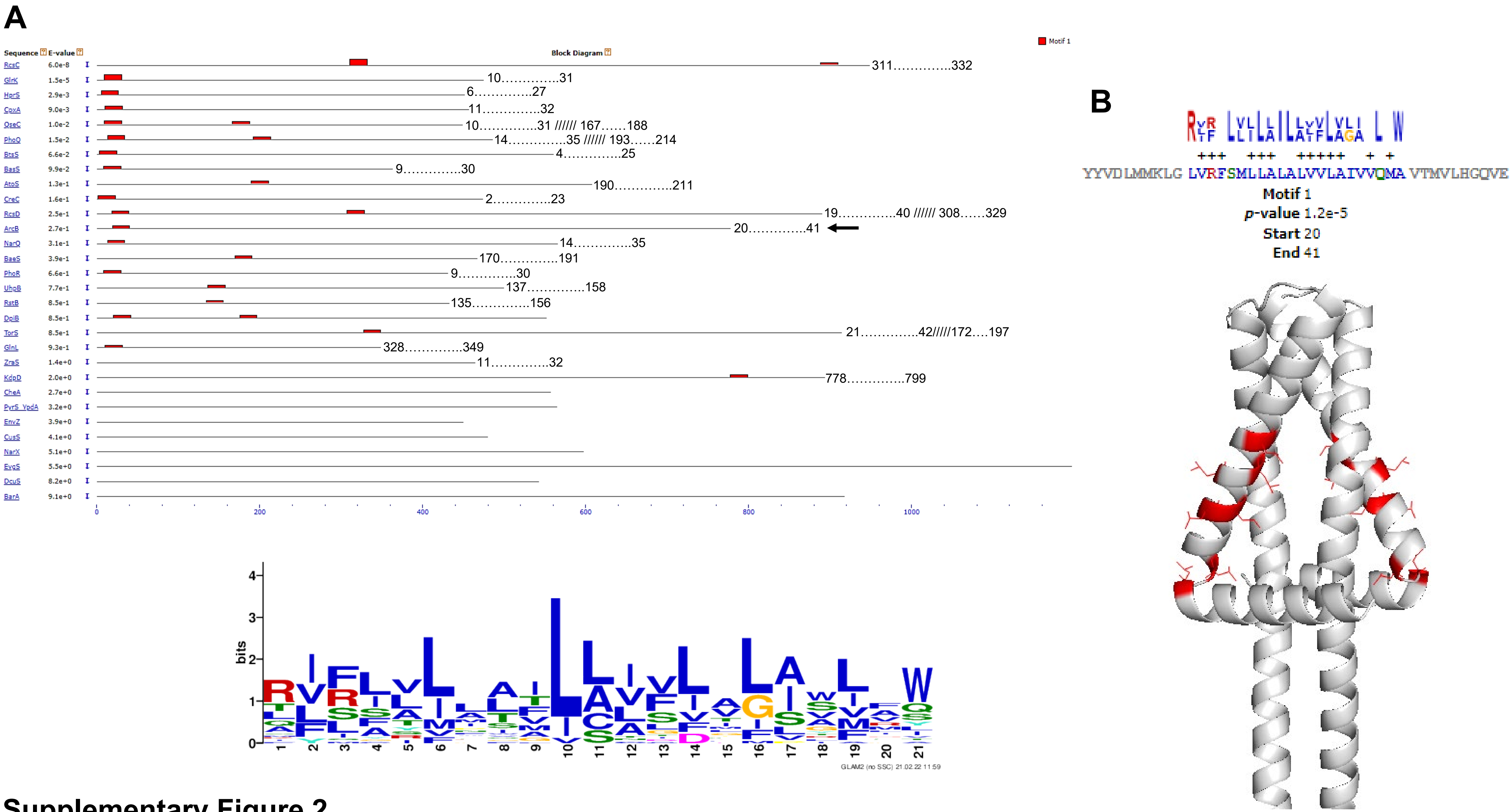

Supplementary Figure 2

1 MKQIRLLAQY YVDLMMKLGL VRFSMLLALA LVVLAIVVQM AVTMVLHGQV 50

51 ESIDVIRSIF FGLLITPWAV YFLSVVVEQL EESRQRLSRL VQKLEEMRER 100

1 11

101 DLSLNVQLKD NIAQLNQEIA VREKAEAELQ ETFGQLKIE**I KEREETQIQL** 150

12 61

151 **EQQSSFLRSF LDASPDLVFY RNEDKEFSGC NRAMELLTGK SEKQLVHLKP** 200

62 111

201 **ADVYSPEAAA KVIETDEKVF RHNVSLTYEQ WLDYPDGRKA CFEIRKVPYY** 250

112 140

251 **DRVGKRHGLM GFGRDITERK RYQDALERAS** RDKTTFISTI SHELRTPLNG 300

301 IVGLSRILLD TELTAEQEKY LKTIHVSAVT LGNIFNDIID MDKMERRKVQ 350

351 LDNQPVDFTS FLADLENLSA LQAQQKGLRF NLEPTLPLPH QVITDGTRLR 400

401 QILWNLISNA VKFTQQGQVT VRVRYDEGDM LHFEVEDSGI GIPQDELDKI 450

451 FAMYYQVKDS HGGKPATGTG IGLAVSRRLA KNMGGDITVT SEQGKGSTFT 500

501 LTIHAPSVAE EVDDAFDEDD MPLPALNVLL VEDIELNVIV ARSVLEKLGN 550

551 SVDVAMTGKA ALEMFKPGEY DLVLLDIQLP DMTGLDISRE LTKRYPREDL 600

601 PPLVALTANV LKDKQEYLNA GMDDVLSKPL SVPALTAMIK KFWDTQDDEE 650

651 STVTTEENSK SEALLDIPML EQYLELVGPK LITDGLAVFE KMMPGYVSVL 700

701 ESNLTAQDKK GIVEEGHKIK GAAGSVGLRH LQQLGQQIQS PDLPAWEDNV 750

751 GEWIEEMKEE WRHDVEVLKA WVAKATKK 778

Supplementary Figure 3

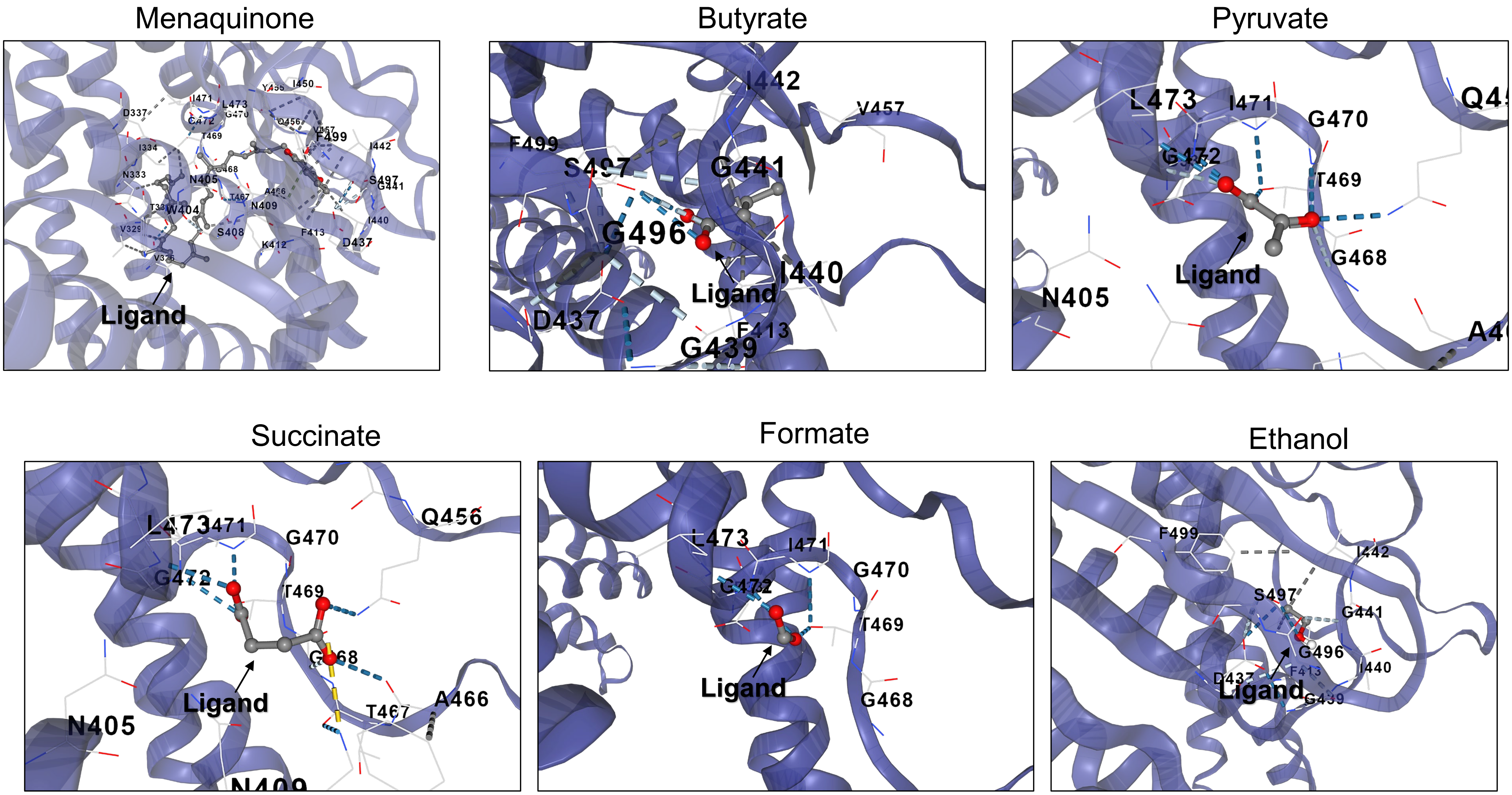

Supplementary Figure 4

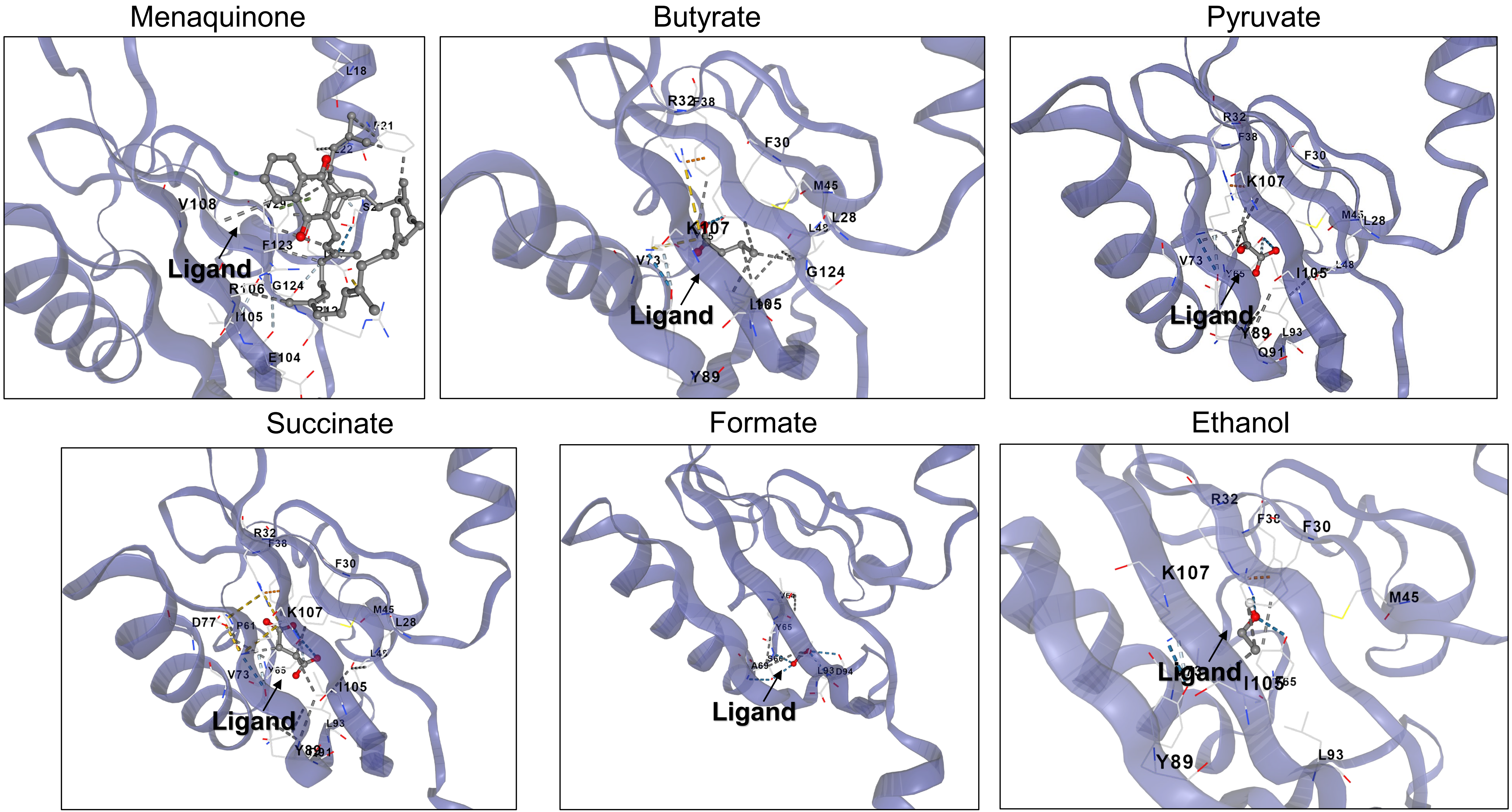

Supplementary Figure 5

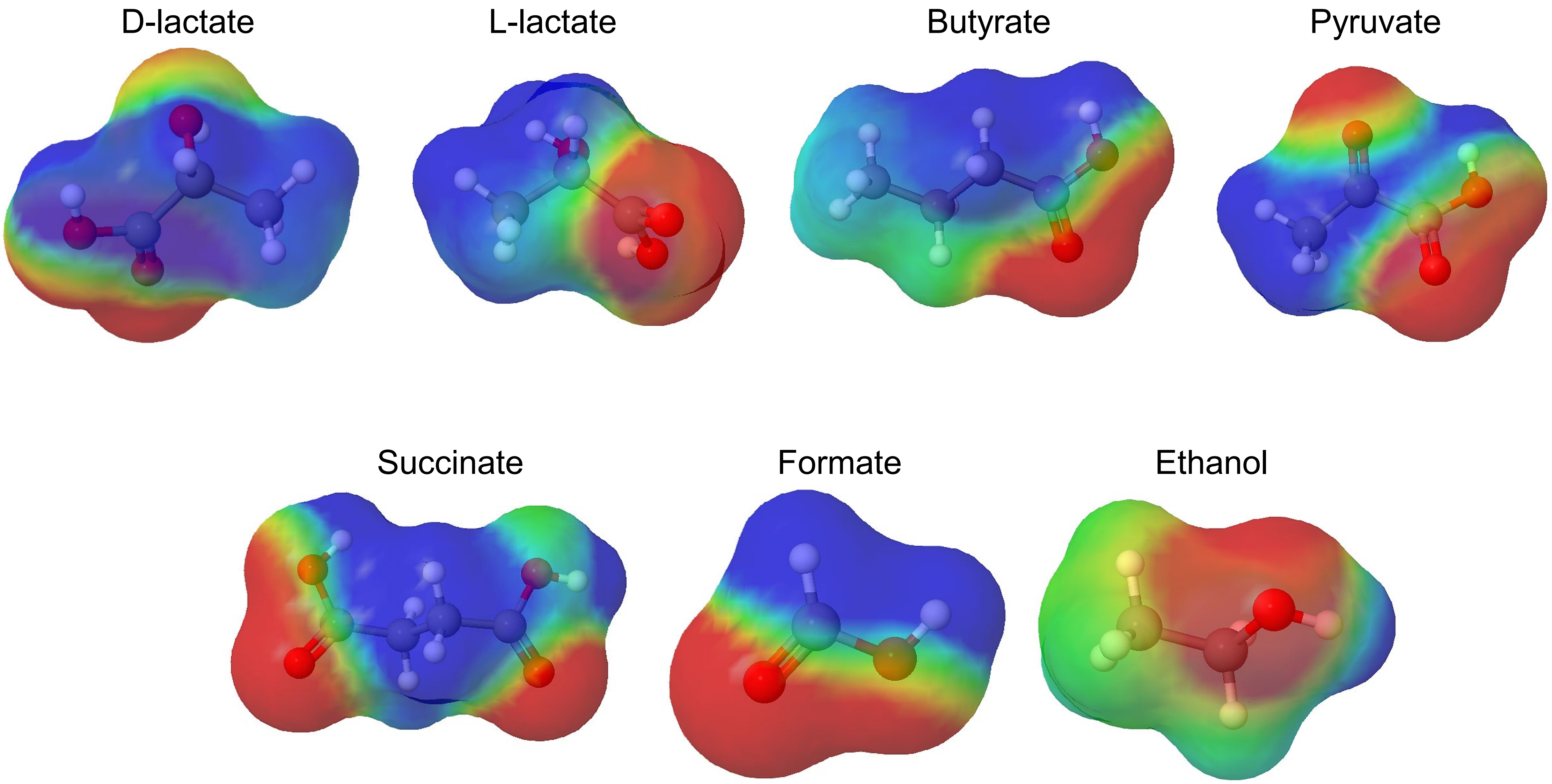

Supplementary Figure 6

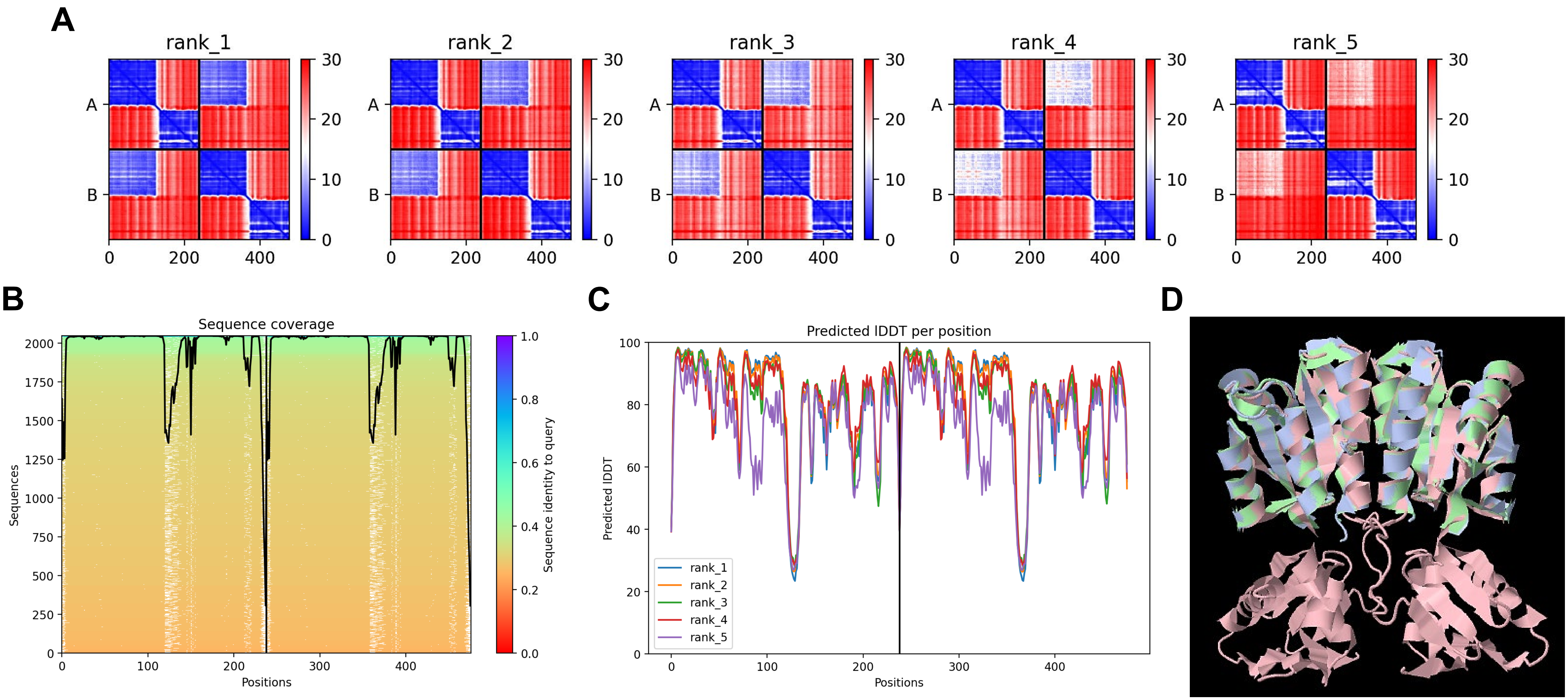

Supplementary Figure 7

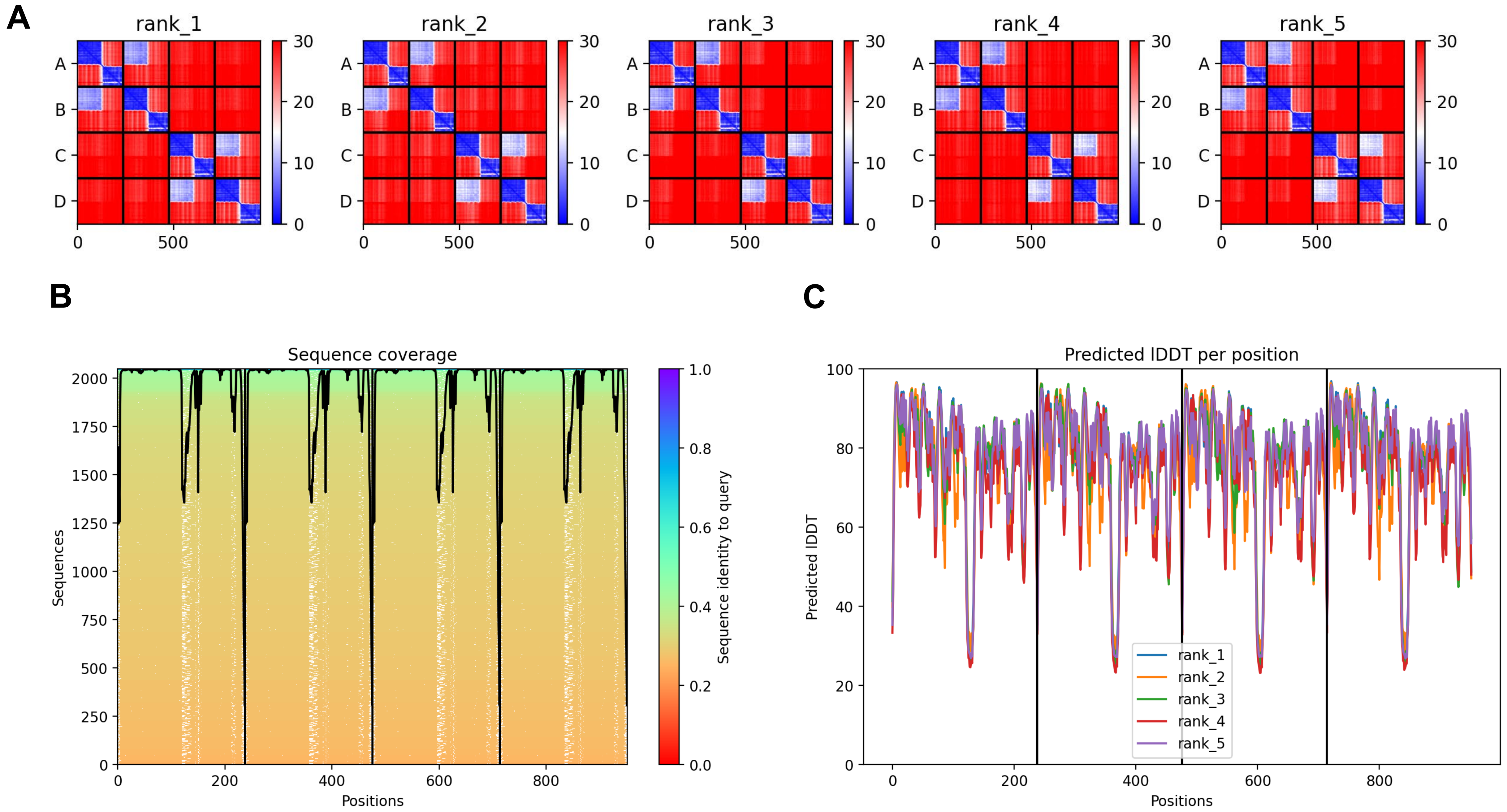

Supplementary Figure 8

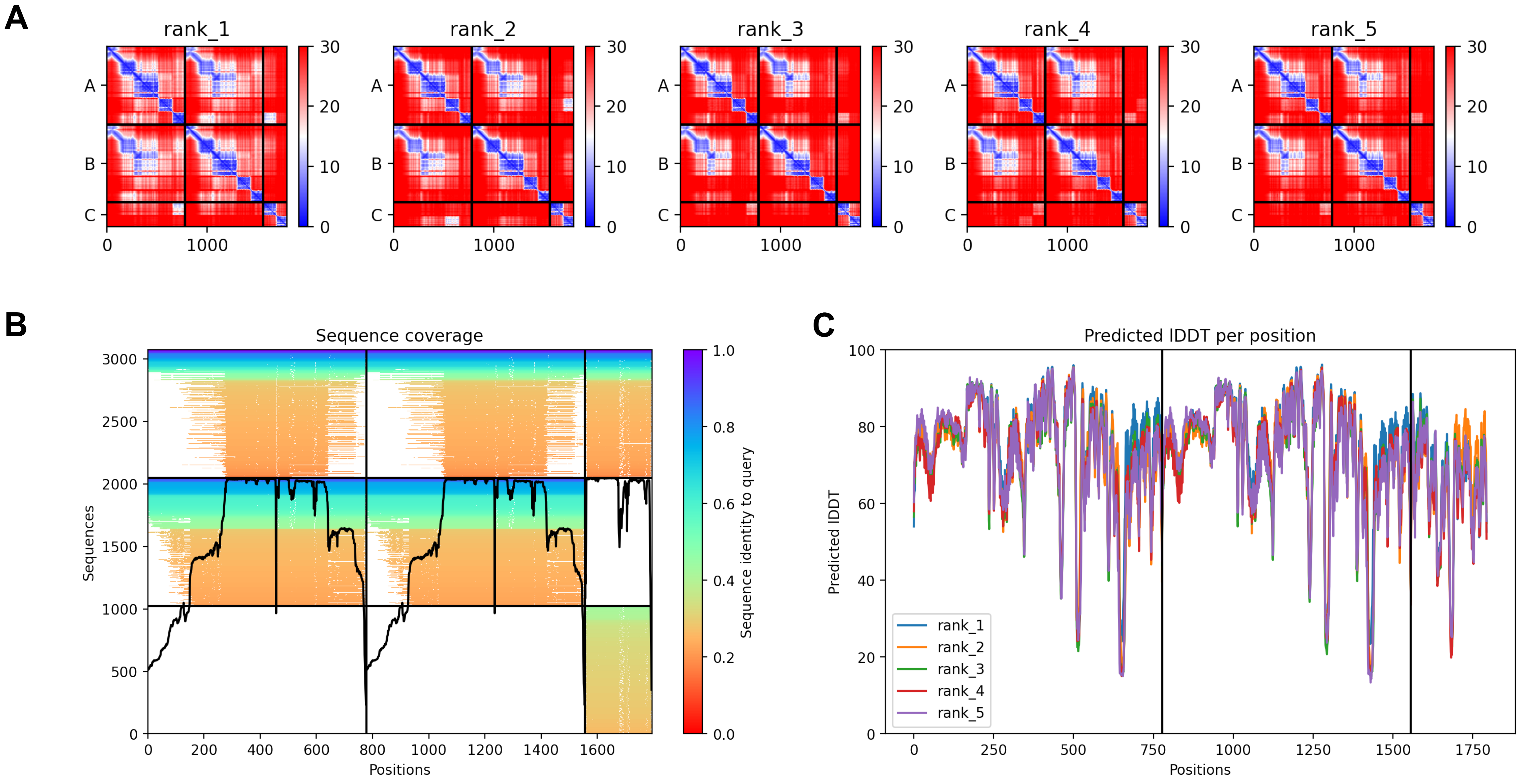

Supplementary Figure 9

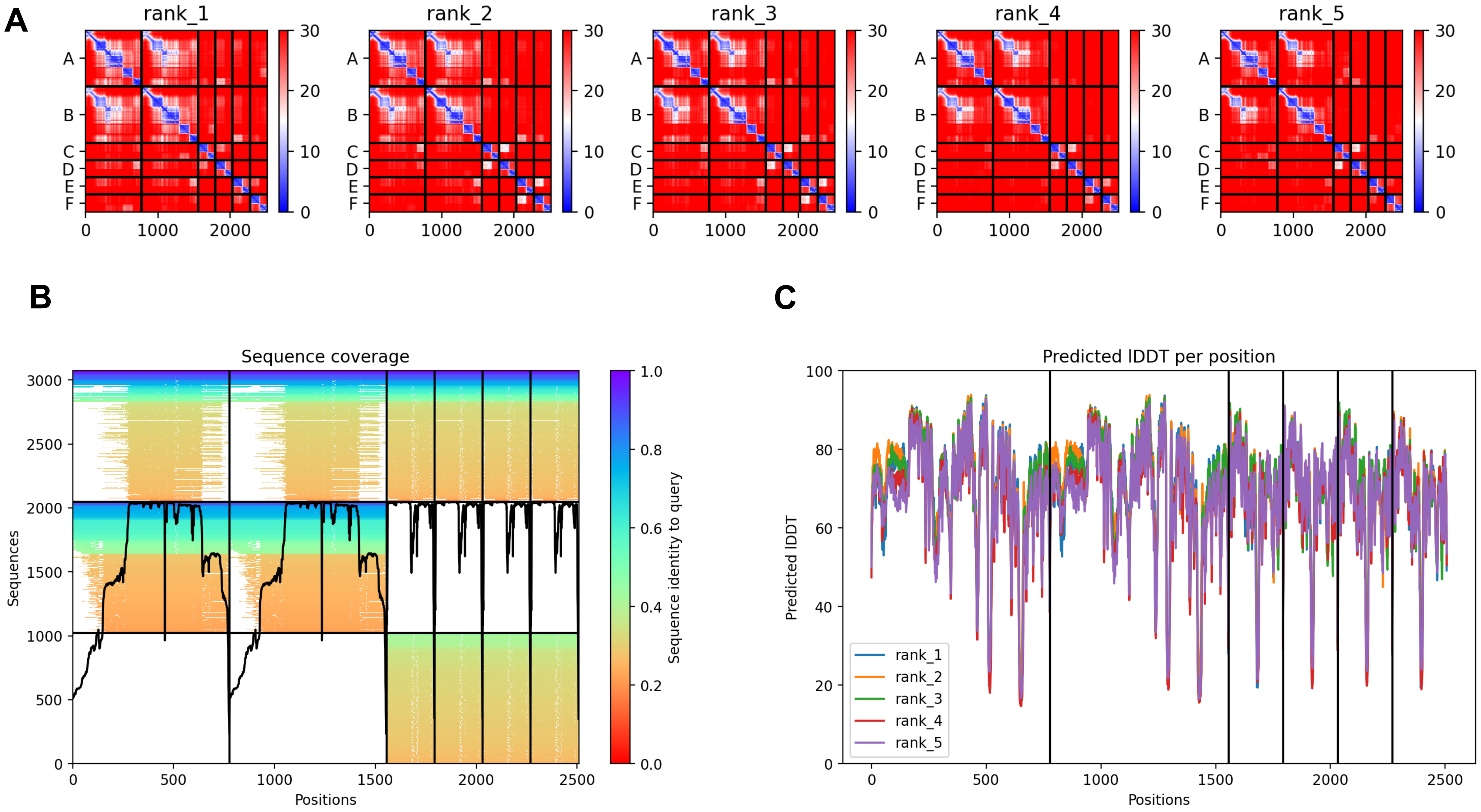

Supplementary Figure 10

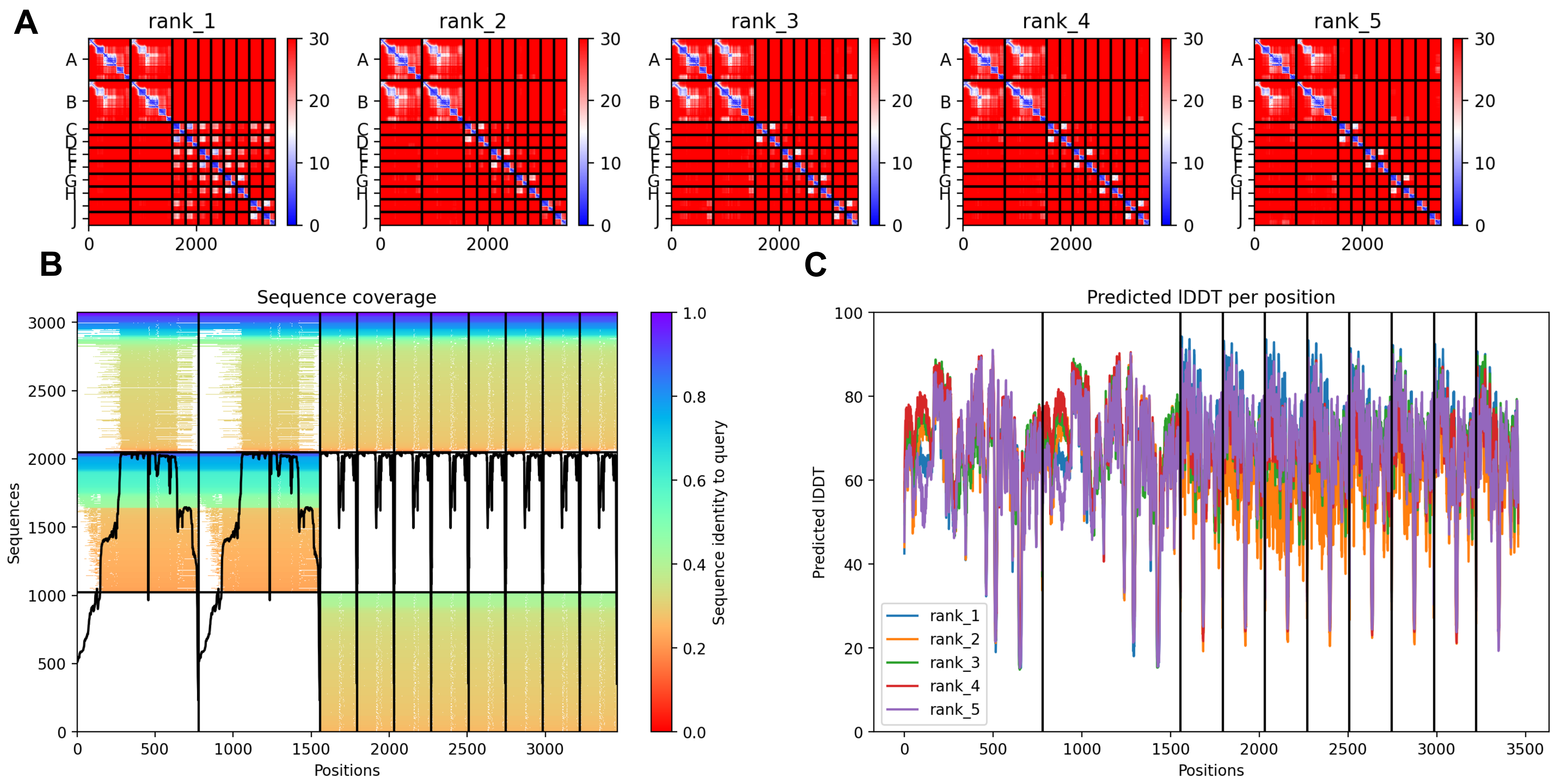

Supplementary Figure 11

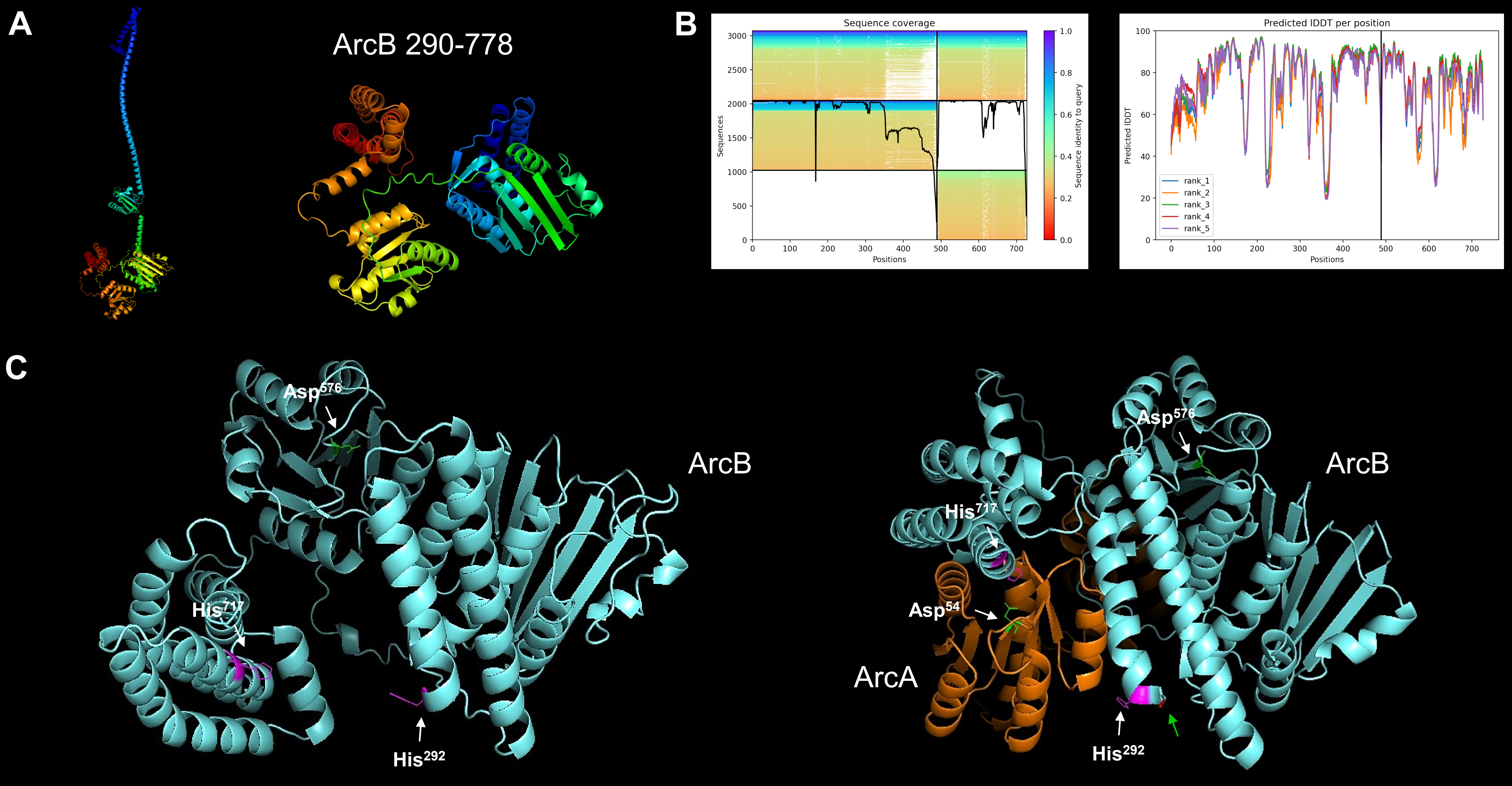

Supplementary Figure 12

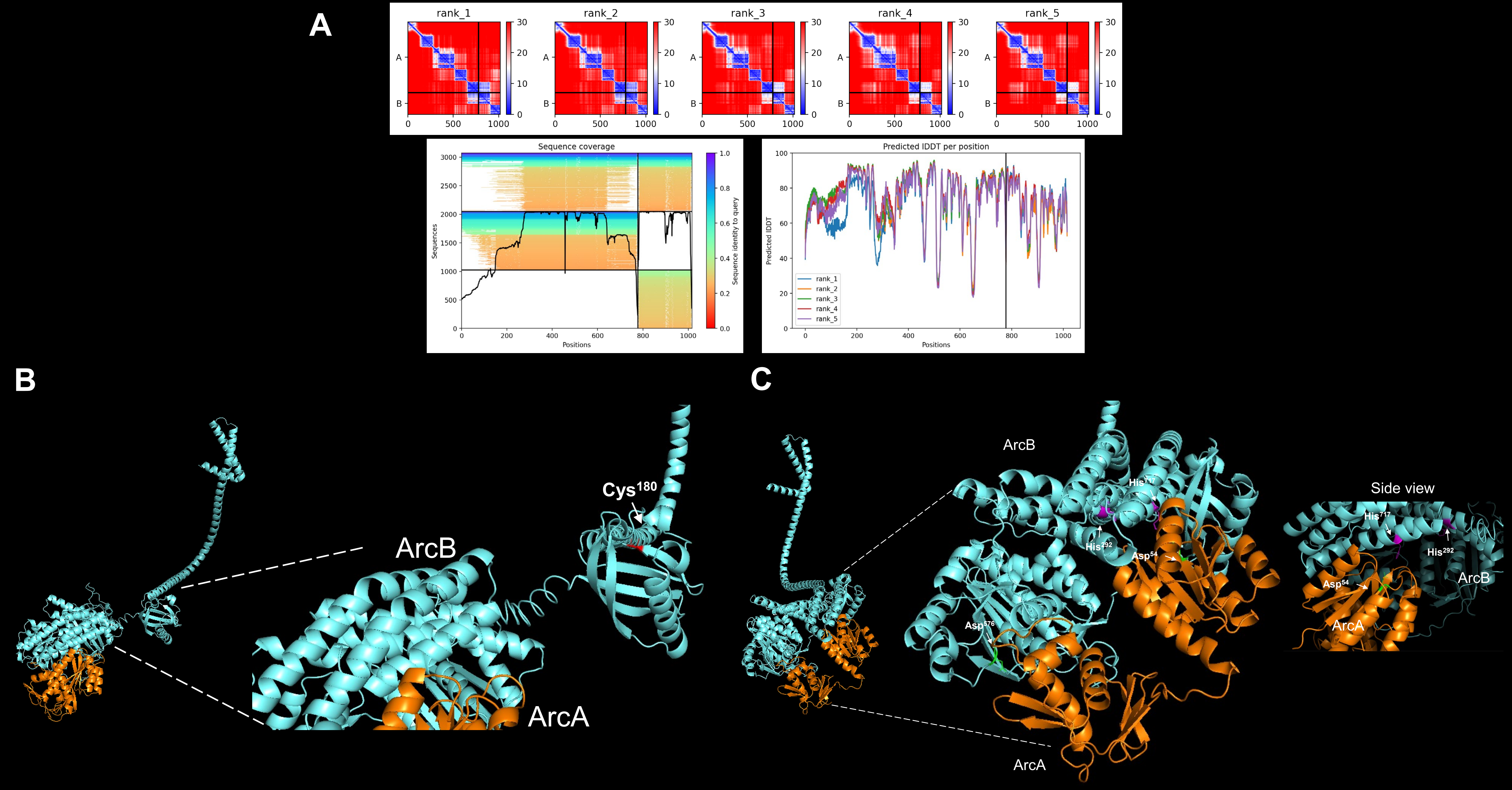

Supplementary Figure 13
