# Supplementary figures and images for: Theoretical study of ArcB and its dimerization, interaction with anaerobic metabolites, and activation of ArcA
Source: PeerJ. 2023 Oct 13;11:e16309. doi: 10.7717/peerj.16309 (PMC10578306; doi:10.7717/peerj.16309)

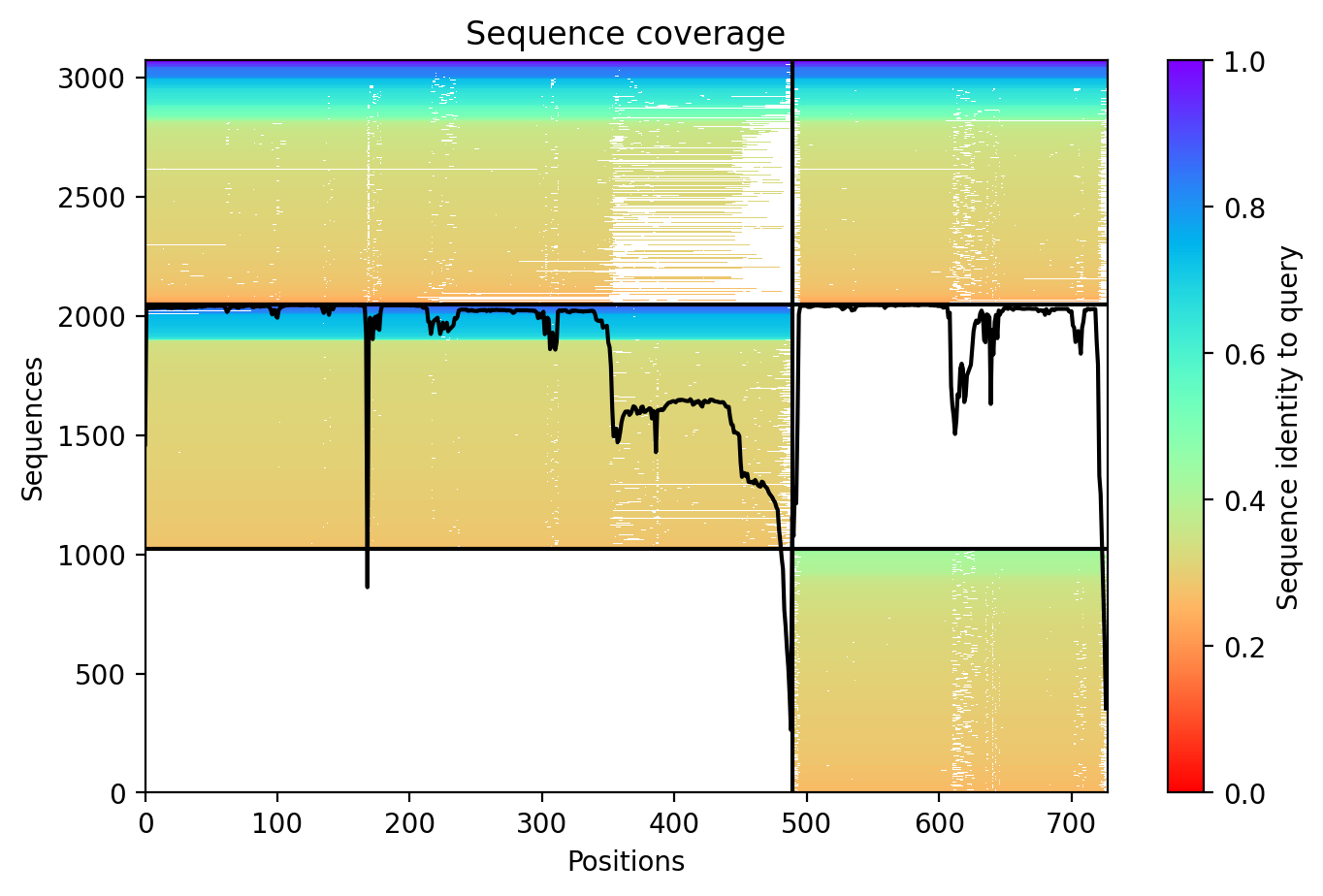

Supplement: Supplemental Information 2 [file peerj-11-16309-s002.zip › Supplementary Files/ArcA and ArcB conformations/AlphaFold models/dimerArcB280_778withArcA/test_be024/test_be024_coverage.png]

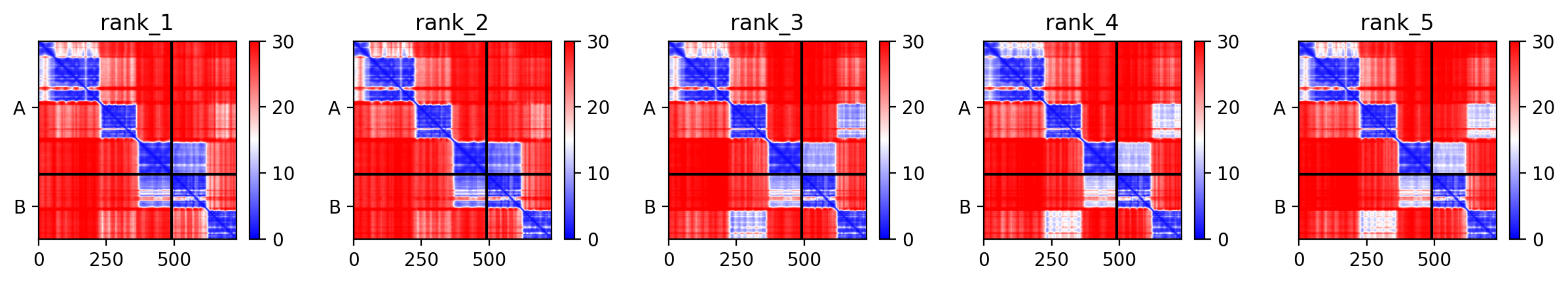

Supplement: Supplemental Information 2 [file peerj-11-16309-s002.zip › Supplementary Files/ArcA and ArcB conformations/AlphaFold models/dimerArcB280_778withArcA/test_be024/test_be024_pae.png]

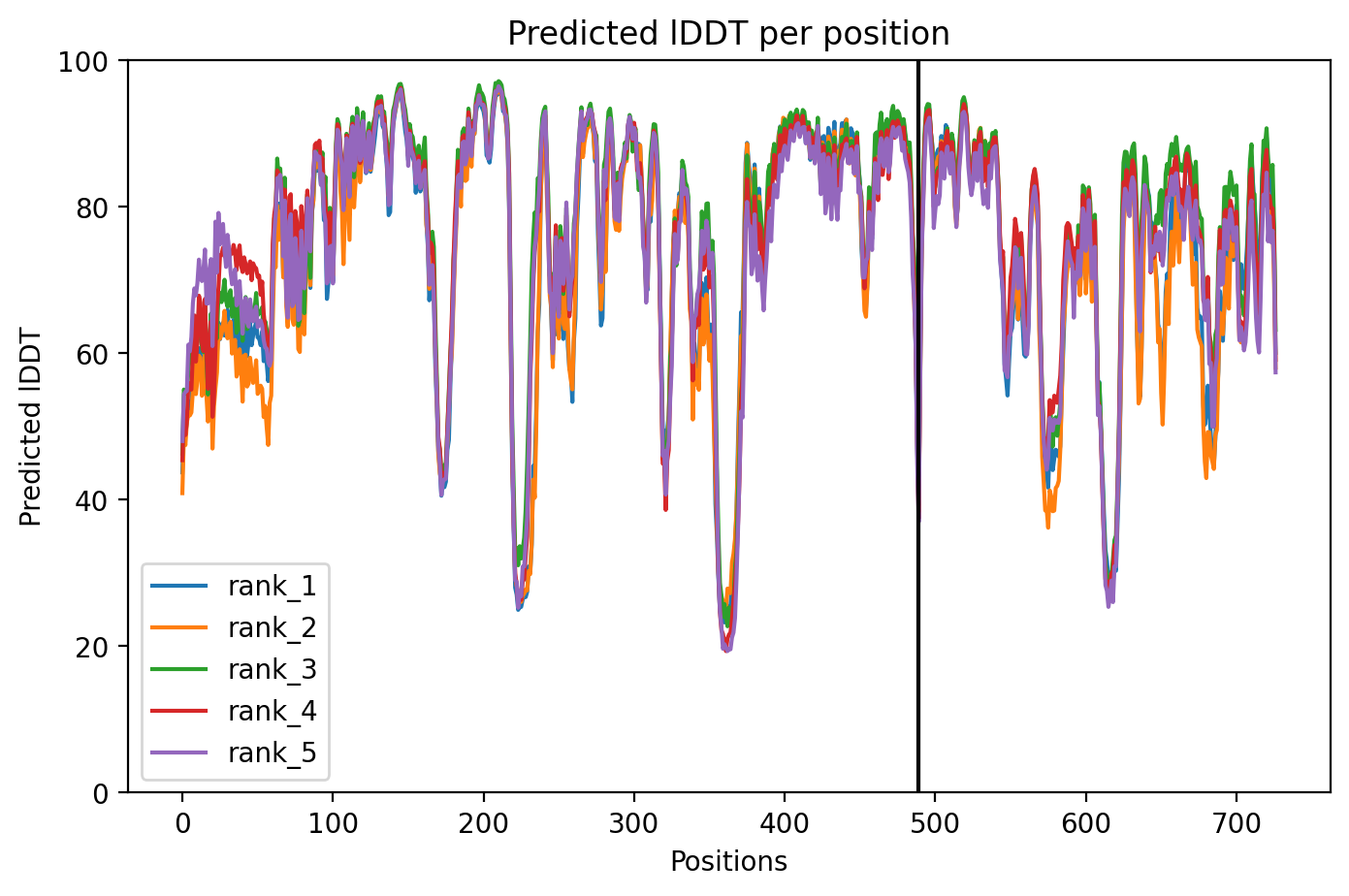

Supplement: Supplemental Information 2 [file peerj-11-16309-s002.zip › Supplementary Files/ArcA and ArcB conformations/AlphaFold models/dimerArcB280_778withArcA/test_be024/test_be024_plddt.png]

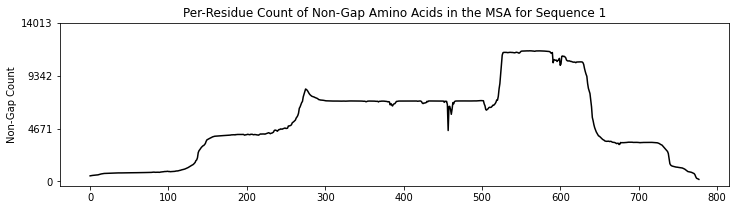

Supplement: Supplemental Information 2 [file peerj-11-16309-s002.zip › Supplementary Files/ArcB dimer/ArcB dimer AlphaFold multimer/coverage monomer.png]

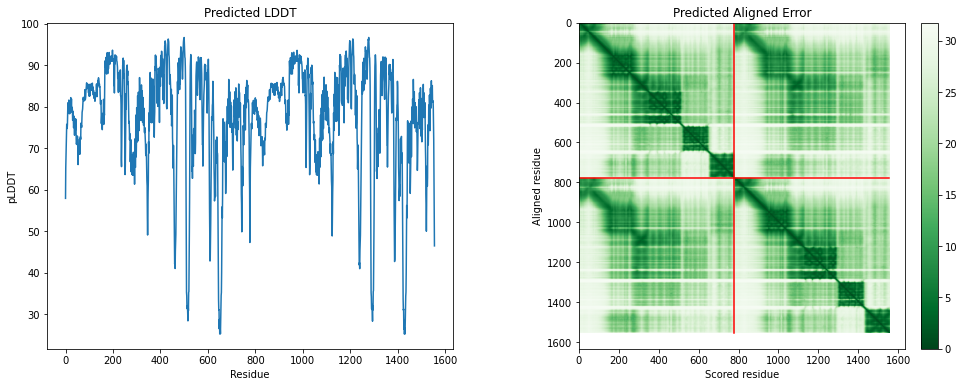

Supplement: Supplemental Information 2 [file peerj-11-16309-s002.zip › Supplementary Files/ArcB dimer/ArcB dimer AlphaFold multimer/pLDDT.png]

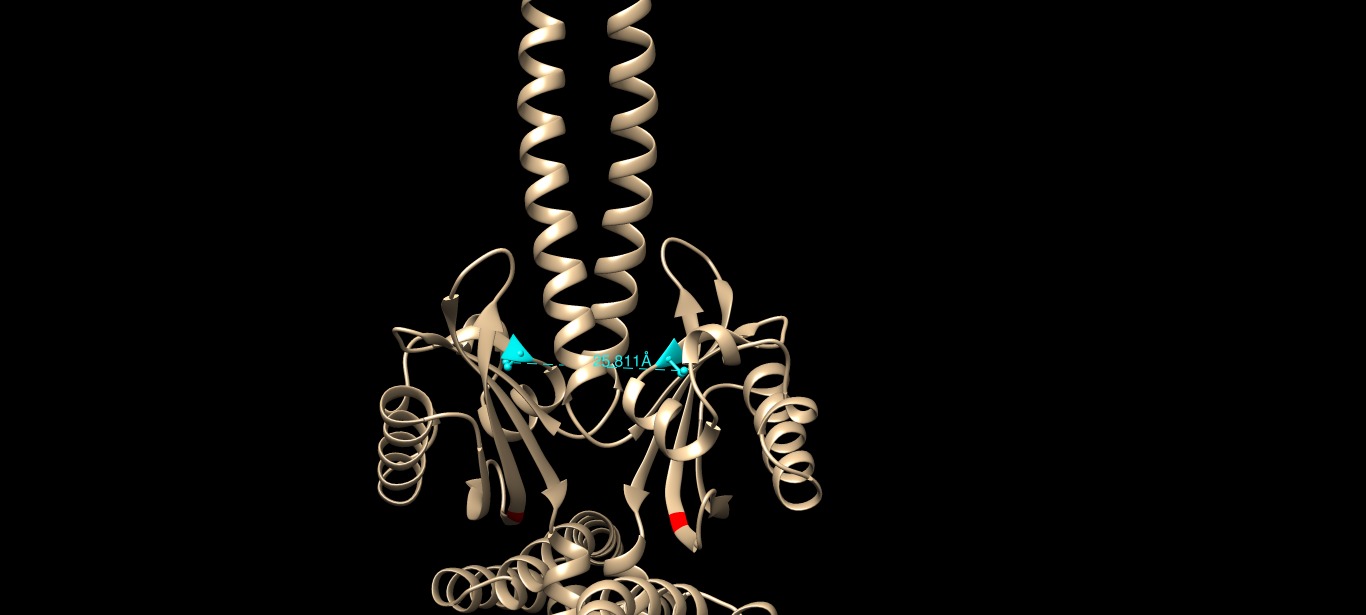

Supplement: Supplemental Information 2 [file peerj-11-16309-s002.zip › Supplementary Files/ArcB dimer/ArcB dimer AlphaFold multimer/prediction/Medicioncyst cyan.tif]

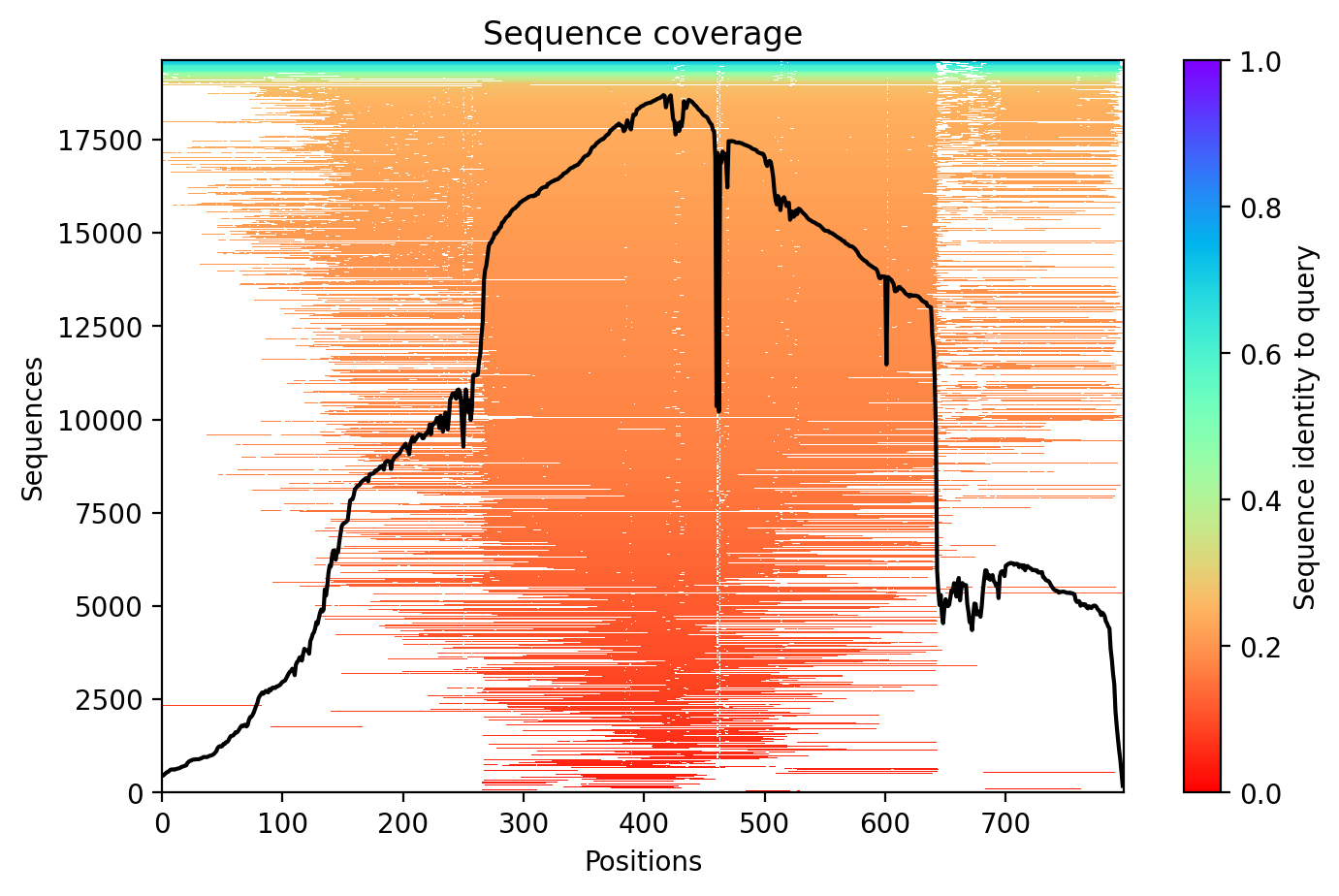

Supplement: Supplemental Information 2 [file peerj-11-16309-s002.zip › Supplementary Files/Type 1 and 2 ArcB sensor kinases models/Type 1/Photobacteriumprofundum_3451c.result/Photobacteriumprofundum_3451c/Photobacteriumprofundum_3451c_coverage.png]

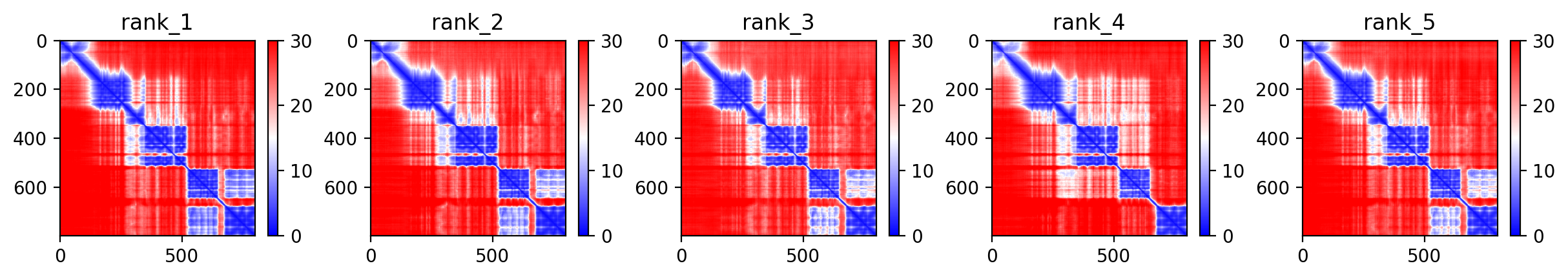

Supplement: Supplemental Information 2 [file peerj-11-16309-s002.zip › Supplementary Files/Type 1 and 2 ArcB sensor kinases models/Type 1/Photobacteriumprofundum_3451c.result/Photobacteriumprofundum_3451c/Photobacteriumprofundum_3451c_pae.png]

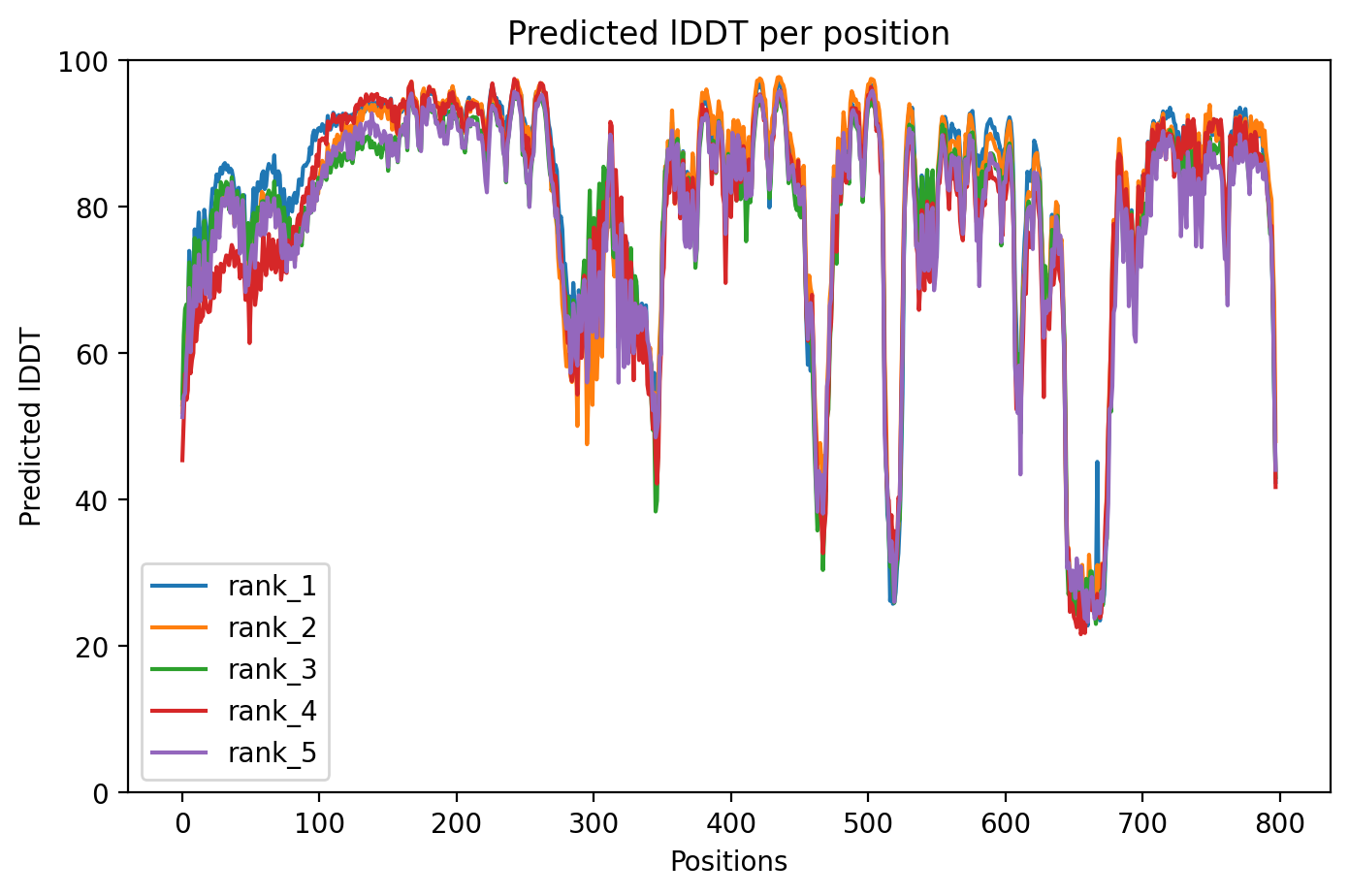

Supplement: Supplemental Information 2 [file peerj-11-16309-s002.zip › Supplementary Files/Type 1 and 2 ArcB sensor kinases models/Type 1/Photobacteriumprofundum_3451c.result/Photobacteriumprofundum_3451c/Photobacteriumprofundum_3451c_plddt.png]

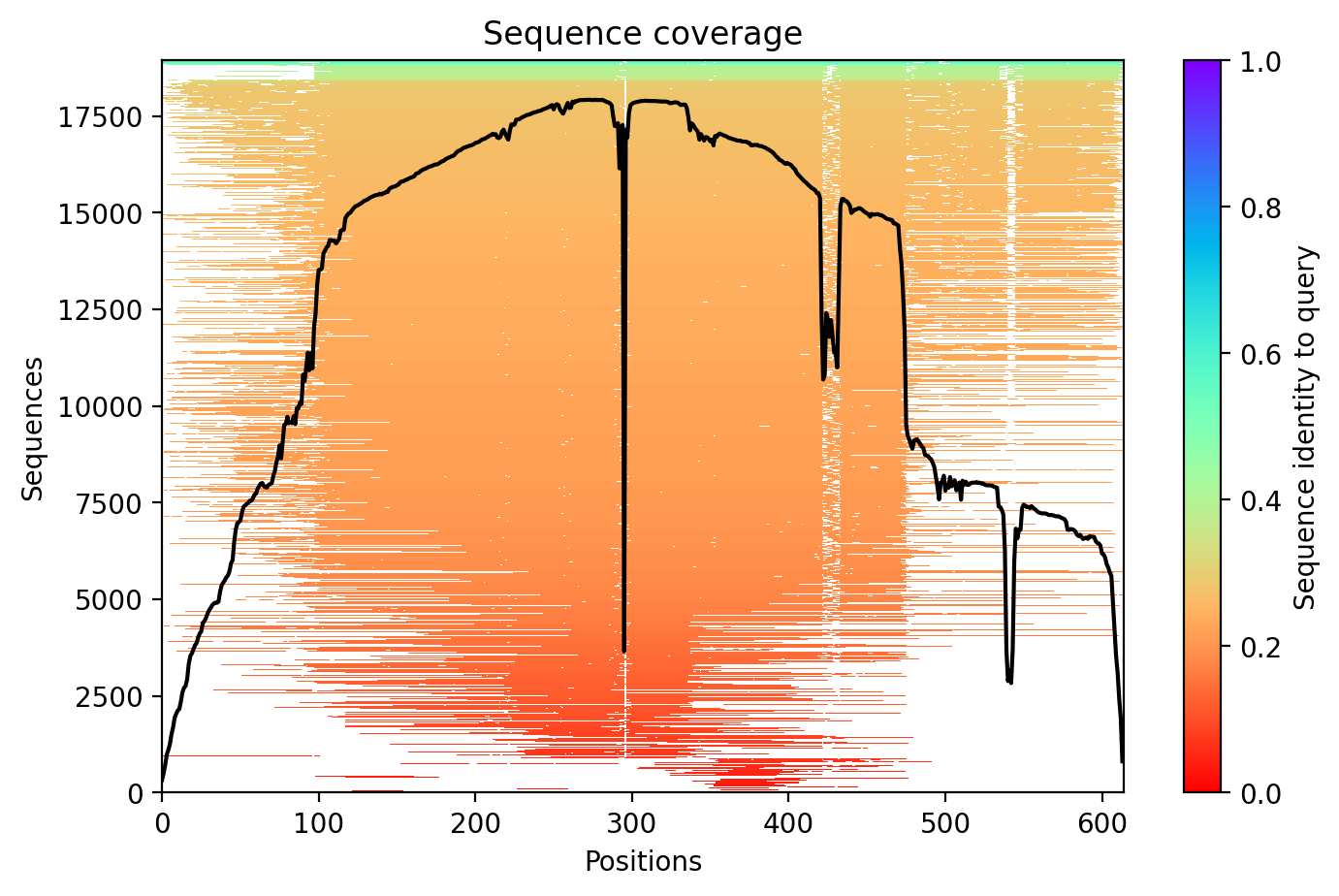

Supplement: Supplemental Information 2 [file peerj-11-16309-s002.zip › Supplementary Files/Type 1 and 2 ArcB sensor kinases models/Type 2/Mannheimia_succiniciproducens_e40f9.result/Mannheimia_succiniciproducens_e40f9/Mannheimia_succiniciproducens_e40f9_coverage.png]

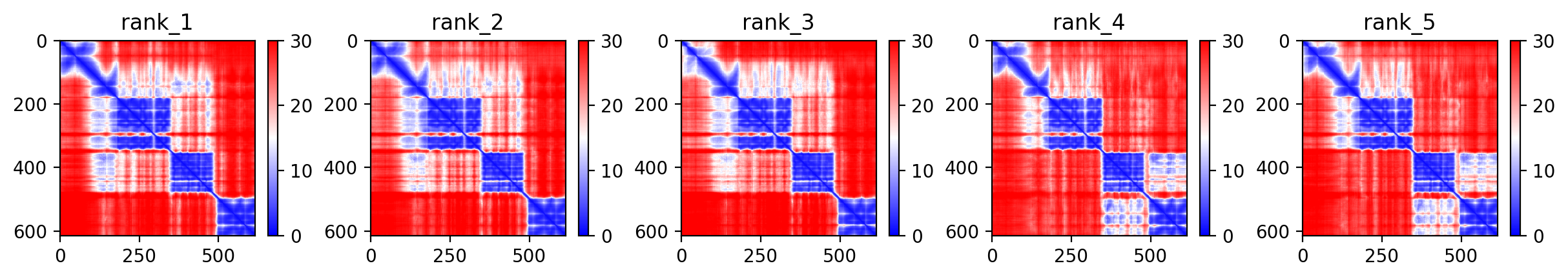

Supplement: Supplemental Information 2 [file peerj-11-16309-s002.zip › Supplementary Files/Type 1 and 2 ArcB sensor kinases models/Type 2/Mannheimia_succiniciproducens_e40f9.result/Mannheimia_succiniciproducens_e40f9/Mannheimia_succiniciproducens_e40f9_pae.png]

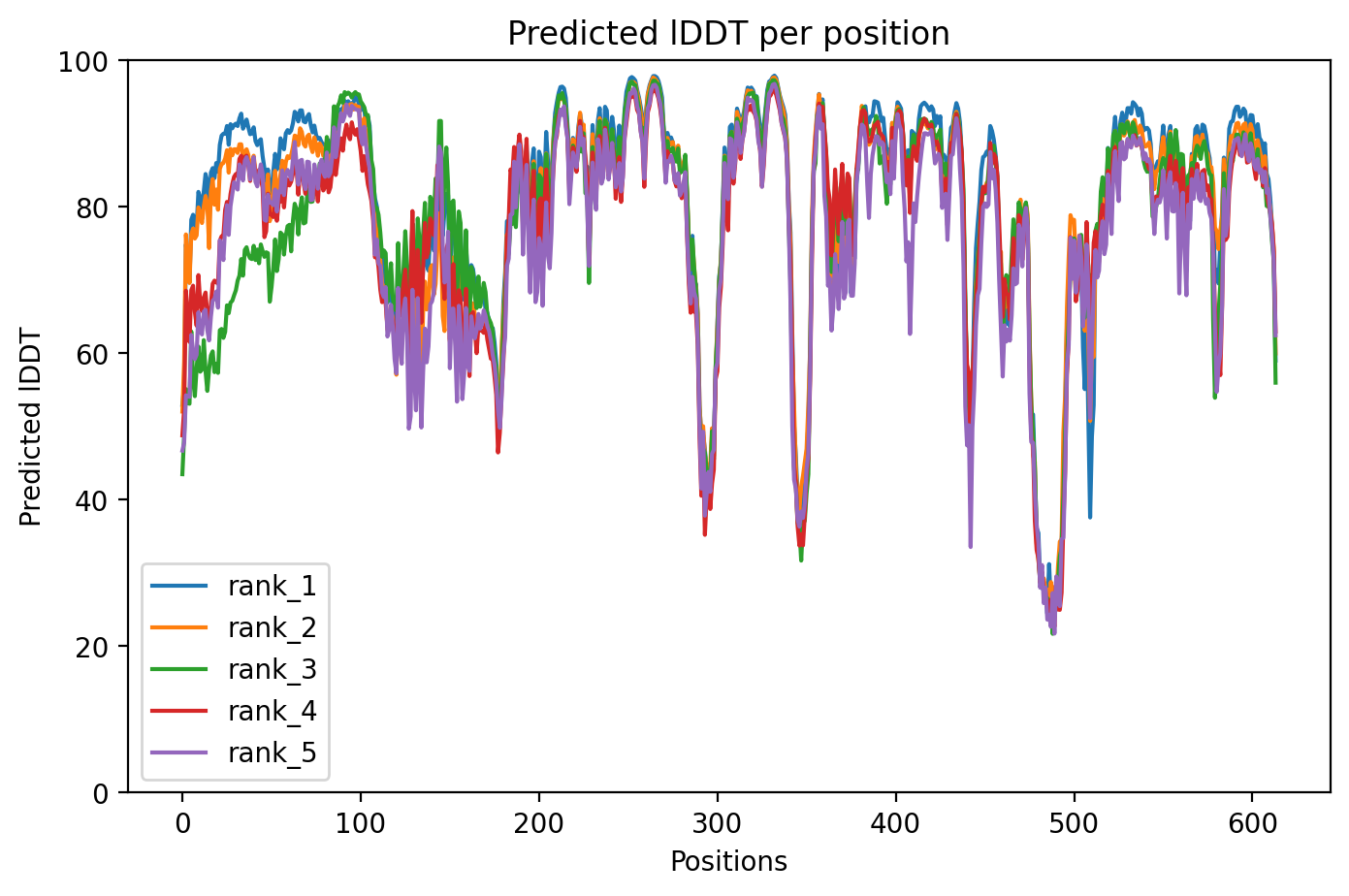

Supplement: Supplemental Information 2 [file peerj-11-16309-s002.zip › Supplementary Files/Type 1 and 2 ArcB sensor kinases models/Type 2/Mannheimia_succiniciproducens_e40f9.result/Mannheimia_succiniciproducens_e40f9/Mannheimia_succiniciproducens_e40f9_plddt.png]
